# Perceptions of self-determination and quality of life among Swedish home care recipients - a cross-sectional study

**DOI:** 10.1186/s12877-019-1145-8

**Published:** 2019-05-24

**Authors:** Karin Bölenius, Kristina Lämås, Per-Olof Sandman, Marie Lindkvist, David Edvardsson

**Affiliations:** 1 Department of Nursing, Umeå University, 90187 Umeå, Sweden; 2 Division of Caring Sciences, Depart Department of Neurobiology, Care Sciences and Society (NVS), Karolinska Institutet, Stockholm, Sweden; 3 Department of Epidemiology and Global Health, Umeå University, Umeå, Sweden; 4 School of Nursing and Midwifery, La Trobe University, Melbourne, Australia

**Keywords:** Aged care, Health, Home care services, Nursing care, Older adults, Older people, Self-determination, Quality of life

## Abstract

**Background:**

It is acknowledged that preservation of self-determination is very important in order for older adults to experience good quality of life, but to what degree and in what areas people receiving help from home care service experience self-determination is unknown. Few studies have examined the perception of self-determination in relation to quality of life among older adults living at home with help from home care services. Thus, the aim of this study was to explore perceptions of self-determination among older adults living at home with the support of home care services, and to test whether older adults who perceive a higher degree of self-determination also feel they have a better quality of life.

**Methods:**

This cross-sectional study was conducted in one municipality in northern Sweden. A total of 134 older adults (≥ 65 years) were included. Data were collected by means of a survey including questionnaires about background characteristics, self-determination, and health-related quality of life. Descriptive statistics regarding background characteristics for groups with high and low self-determination respectively were presented and the differences between the groups were analyzed using the Chi-square test and the Mann-Whitney U test.

**Results:**

Our main finding shows that the majority of older adults with support from home care services experience self-determination in the dimensions use of time, and self-care. However, a wide variation was found in self-reported self-determination in all dimensions. Results also show that the group with higher self-reported self-determination also reported a greater degree of experienced quality of life in comparison with the group with lower self-reported self-determination.

**Conclusions:**

In line with earlier research, our results found a positive relation between self-determination and quality of life. The results are relevant for the care of older adults and indicate a need of further research. The results presented in this paper could serve as a guide when planning for improved self-determination among older adults in home care service.

**Trial registration:**

NCT02846246.

## Introduction

It is reasonable to hypothesize that older adults living at home with support from home care services (HCS) have a better quality of life (QoL) if their own expressed needs regarding care and service are met. A definition of QoL by the WHO is individuals’ perception of their position in life in the context of the culture and value systems in which they live and in relation to their goals, expectations, standards and concerns [1]. Studies from other areas, for example those living with type 2 diabetes [2] and pain relief [3], reinforce that having self-determination regarding decisions about one’s care contributes to QoL. A Finnish qualitative study showed that two thirds (approx. 943 older adults) of a sample of 1405 older adults expressed a desire to pass on before reaching 100 years of age, with 24% stating due to being afraid of losing health, and 10% stating because of fears of losing self-determination [4]. Based on this, it seems reasonable to hypothesise that increased self-determination among older adults who receive HCS makes up an important part of their QoL.

Self-determination in relation to QoL in HCS has not been widely studied in the nursing literature. Thus, it seems important to expand the knowledge base regarding factors concerning self-determination and self-determination in relation to QoL for older adults. Increased knowledge in this area can help care managers and researchers to develop new interventions that facilitate QoL. This paper extends the available knowledge with regard to self-determination among older people living at home with daily home care and explores whether groups with more as opposed to less self-determination differ in terms of QoL.

## Background

Autonomy is often used as a synonym for self-determination but is more closely linked to the idea of individuals taking their own decisions without being influenced by others. This might make it problematic to use the concept of autonomy in connection with a person dependent on help and with decisions taken together with others [5]. Autonomy defined in another way describes self-determination as a central aspect of autonomy, as the capacity to act and decide in accordance with one’s own free wishes [6]. Self-determination has also been defined as a process in which a person has control and ethical/ legal rights [7], and as the capacity to make personal choices, irrespective of the person’s ability to accomplish those choices [8]. In this paper we assess self-determination, using the Impact of Participation and Autonomy of Older People (IPA-O) (see method section) where the concept of self-determination is used synonymously with decisional autonomy, [9] meaning the ability to make decisions and to execute choices without external restraint [8].

Practising self-determination has been shown to have an impact on older adults receiving HCS in several ways and a variety of experiences have been described. Feeling safe, having an opportunity to influence and be involved in decisions [10], being free to choose and in control of everyday life and having ones’ needs met [11] are examples of impacts experienced. In contrast, people with little self-determination have described a sense of lack of control, being careful about taking risks, a need to retreat into an isolated world [12], and insecurity [13]. Thus, it is reasonable to believe that promoting self-determination will increase overall QoL.

To promote self-determination among older adults, both the policy and organisation of HCS need to confirm their wishes and needs [5]. For example, suggestions to be incorporated into future care models include having a culture of care that focuses on respect for the older adult [14], where leaders maintain continuity and work to improve nurses’ attitudes [15]. To further promote self-determination and a sense of being respected among older adults, staff need to be flexible in their provision of daily care whenever a problem arises [11], and strive to establish meaningful relationships between the older person, colleagues and leaders within the organisation [5, 14, 16–18]. A trusting and positive relationship is described as meaningful in enabling involvement in daily care and it’s planning [11, 16, 19], and a positive relationship might motivate the older person to share information about personal issues and wishes [11, 20]. In contrast, one review [5] found that organisations which allocated time and care from the perspective of the organisation rather than the older person’s wishes and needs had a negative influence on the self-determination of older adults. In addition, the functional capacities of older adults can impede self-determination [5]. A Swedish study reported that self-determination among people living at home with HCS support, who were dependent in both P-ADL and I-ADL, showed significantly less self-determination (p⩽ 0.05) than people who were independent [21].

Both international [11, 15, 22] and Swedish studies [23, 24] report limited possibilities for older adults to practice self-determination concerning the content of care in HCS. The international studies [11, 15, 22] describe older adult’s self-determination as being influenced by, for example, the healthcare staff’s individual working methods and the multifaceted, hierarchical and unpredictable healthcare structure. The Swedish studies [23, 24] report that the possibilities for older adults to be involved in decisions and to have any real influence over the content of care are limited. This is surprising, because, in the literature, older adults whose expressed home care needs are met have reported significantly higher levels of life satisfaction, lower levels of loneliness and perceived life stress than elderly people whose needs are not met [25].

There are studies which describe the importance of self-determined decisions for improving QoL regarding care in other areas, such as diabetes type 2 [2, 26] and, pain relief [3]. However, to our knowledge, there is no study that explores self-determination in relation to QoL among older adults living at home with support from HCS.

QoL is a broad concept affected in a complex way by the person’s physical health, psychological state, level of independence, social relationships and their relationship to salient features of their environment [26]. Studies show that QoL among people aged 75 years and older with care needs is not affected by the type of helper [27] but that social isolation [28, 29] and the extent of the help influenced it negatively. Among adults aged 75 years and older living at home without home care, it has been found that 85–94% rated their QoL as being good or very good compared to only 64–74% of those living at home and receiving home care [29]. There is a need of more knowledge regarding self-determination in relation to health-related QoL among older adults receiving HCS [11].

### Rationale

It is acknowledged that preservation of self-determination is very important for older adults to experience good QoL, but to what degree and in what areas people receiving help from HCS experience self-determination is unknown. Searches in databases relevant to the topic of nursing, show that it is essential to practice self-determination and it is reasonable to believe that a greater degree of self-determination influences QoL positively. Few studies have actually explored self-determination as a factor which might influence QoL among older adults living at home with help from HCS. Since the main goal of HCS is to ensure that older adults live a worthwhile life with a sense of wellbeing [30] and in order to improve QoL among older adults who receive HCS [31] it is essential to explore any possible relationship between self-determination and QoL. Such knowledge will make an important contribution to understanding how quality aged care can be created. Thus, the aim of this study was to explore perceptions of self-determination among older adults living at home with the support of home care services, and to test whether those older adults who perceive a higher degree of self-determination also have a higher health-related QoL.

## Methods

### Design

We conducted a cross-sectional study using three questionnaires, *Impact on Participation and Autonomy, the Nottingham Health Profile,* and the *EQ-5D-5 L.* The study is part of a larger intervention study aiming to evaluate: the effects and meaning of a person-centred and health-promoting HCS intervention on QoL; thriving and satisfaction with care in older adults; caregiver strain, informal caregiving engagement and satisfaction with care among relatives; and on job satisfaction and stress of conscience among home care staff [32]. In this study, HCS is interpreted widely and concerns the kind of help given by health professionals in the homes of older adults, such as shopping, cleaning, cooking, feeding, hygiene, showering etc.

### Context and participants

This study was conducted in HCS section in one municipality in northern Sweden, where HCS is provided by municipalities as a universal service [33]. The municipality in this study, comprised 10 districts in total. Older adults (*n* = 356) from one geographical HCS district were invited to participate in a larger intervention study based on a power calculation where *N* = 207 will provide 5% power at the 0.05 significance level. In total, 163 participants agreed to be part of the intervention study. A sub-sample of 134 (82%) from the intervention study who had answered questions about self-determination were included in the present study. Inclusion criteria for this study were; aged 65 years or older, living at home and receiving HCS with at least two visits per month, able to speak Swedish, be strong enough to answer the questionnaires used in the study, and be judged by staff/ family member to have a good cognitive status. A good cognitive status means a person who is judged to answer questions with credibility.

### Data collection

Data were collected by means of a survey including questionnaires about background characteristics, self-determination, health-related QoL (Table 1). All included questionnaires have a published track record of satisfactory validity and reliability [9, 34–37]. Older adults who struggled to write or read were offered support from a retired Registered Nurse trained to support completion of the questionnaires. The Registered Nurse worked with the participants individually and ticked the boxes in the questionnaires, one at a time, in accordance with their answers. Background characteristics such as the participants’ degree of dependence in activities of daily living (ADL) were collected. The ADL assessment comprised a total of 15 items divided in two domains, instrumental ADL [34] and personal ADL [35]. Instrumental ADL includes questions about shopping for food, heavy housekeeping, and ability to handle finances etc. with options that were scored on a 3-, 4- or 5-point Likert scale. Personal ADL includes questions about showering, dressing, and eating etc. with options scored on a 3-point Likert scale. High scores indicate independence. The options in both domains were dichotomized before analysis. For both domains, scoring levels indicating a need for help to any extent were scored as 0 and total independence as 1.

**Table 1:** Characteristics of the participants

Characteristic	Participants	Higher self-determination	Lower self-determination	*p*
	*n* = 134	*n* = 65	*n* = 69	
Sex				.930^a^
Female n (%)	84 (63)	40 (62)	44 (64)	
Male n (%)	50 (37)	25 (38)	25 (36)	
Age (Year)				.246^b^
Median (Q1; Q3)	83 (77; 88)	84 (79; 89)	83 (74; 88)	
Range	65–100	66–97	65–100	
Help from HCS n (%)				.788^a^
< 1 year	25 (20)	13 (22)	12 (19)	
1–10 years	85 (69)	43 (71)	42 (66)	
≥ 10 years	14 (11)	4 (7)	10 (15)	
Not answered *n* = 10				
Number of home visits n (%)				.622^a^
1–2 visits/ week or 1 every fourteen days	39 (31)	21 (35)	18 (27)	
3–6 times each week	12 (10)	5 (8)	7 (11)	
Each day	23 (18)	9 (15)	14 (21)	
Twice each day	23 (18)	13 (22)	10 (15)	
Three or more each day	29 (23)	12 (20)	17 (26)	
Not answered *n* = 8				
Housing n (%)				**.034** ^a^
House	25 (19)	17 (26)	8 (12)	
Apartment	108 (81)	48 (74)	60 (88)	
Not answered *n* = 1				
Educational background n (%)				
Elementary school	63 (48)	31 (48)	32 (47)	.738^a^
Secondary school	47 (36)	21 (33)	26 (38)	
University	22 (16)	12 (19)	10 (15)	
Not answered *n* = 2				
Country of birth n (%)				.272^a^
Sweden	115 (86)	58 (89)	57 (83)	
Other countries	19 (14)	7 (11)	12 (17)	
Swedish is the mother tongue n (%)				.783^a^
Yes	124 (93)	61 (94)	63 (93)	
No	9 (7)	4 (6)	5 (7)	
Not answered *n* = 1				
Instrumental activities in daily living n (%)				1.000^a^
Dependent	119 (95)	56 (95)	63 (95)	
Independent	6 (5)	3 (5)	3 (5)	
Not answered *n* = 9				
Personal activities in daily living n (%)				.818^a^
Dependent	63 (83)	24 (80)	39 (85)	
Independent	13 (17)	6 (20)	7 (15)	
Not answered *n* = 58				

p = Differences between higher and lower self-determination, ^a^= Chi-square test, ^b^ = Mann-Whitney U Test, Not answered = Internal missing values are not included in the analyses

The IPA-O questionnaire was used to assess self-determination. In IPA-O the concept of self-determination is used synonymously with decisional autonomy [9] meaning the ability to make decisions and execute one’s own choices without external restraint [8]. The IPA-O comprises 22 items covering the dimensions of; “mobility”, four items, “self-care”, five items, “activities at home”, four items, “social relationships”, five items, “use of time”, one item, “financial situation”, one item, “providing help and support for others”, one item, and finally “I live as I want”, one item. Two examples of items in IPA-O are listed; Mobility, “My chances to decide where to get around in my house are good” and Self-care, “My chances to decide to get washed and dressed the way I want are good”. All dimensions with more than one item showed good internal consistency with Cronbach’s alphas between 0.74–0.86. Answers were scored on a five-point Likert scale: totally agree (1), partly agree (2), neither agree nor disagree (3), disagree (4), and totally disagree (5). The IPA-O was found to have good face and content validity [9]. In addition, IPA-O showed moderate or high reliability in all questions except for one item in the dimension mobility that had low reliability. A more detailed description of each item is reported [9]. Because of a mistake in its construction, one item (financial situation) was not included in the questionnaire used. To allow comparison, the answer alternatives were dichotomized [21], before analysis of the older adults’ experience of self-determination. The answer alternative “totally agree” was considered to convey self-determination and the options “agree partly” to “disagree” were considered to express not experiencing self-determination. The total IPA-O showed good internal consistency (Cronbach’s alpha .85) in our study which allowed analysis of associations between self-determination and quality of life by using the total score of the scale. Reported self-determination was dichotomized to higher and lower self-determination, based on the cut-off at the median score of 33 (Q1;25, Q3;40) for the total group. The score ranges from 21, highest degree of perceived self-determination, to 105, lowest degree.

Two questionnaires were used to assess health-related QoL; the *Nottingham Health Profile* (NHP) for psychosocial aspects [36] and the *EQ-5D* for functional aspects by [37]. The NHP comprises 38 items divided into 6 dimensions: energy level, pain, emotional reaction, sleep, social isolation, and physical abilities. Two examples of items in NHP are listed; energy level, “Everything is an effort” and pain, “I’m in constant pain”. Each item is presented as a statement with a Yes/No response and an index of the total score in the dimensions ranges from best (0) to worst (100) possible health. The NHP has been found to be valid [36] and reliable [38]. The *EQ-5D 5 L* comprises two parts, a state-of-health description, which includes 5 items, and a visual analogue scale. The state-of-health description comprises five dimensions: mobility, self-care, usual activities, pain/discomfort, and anxiety/depression. Each dimension is scored on a five-point Likert scale, ranging from no problem (0) to extreme problem (4). The answers to the items are combined in a 5-digit value representing the state of health, which is then given a tariff value that is preference based and derived from a British general population. The tariff value ranges from worse than dead (< 0) to 1 (full health), anchoring dead at 0. The visual analogue scale rates participants’ overall health between endpoints, from worst imaginable health (0) to best imaginable health (100). The EQ-5D has been found to be valid and sensitive to change [37].

### Analyses

#### Statistical analyses

Descriptive statistics regarding background characteristics for groups with high and low self-determination respectively were presented with number and percentages for categorical variables and median and quartiles for quantitative variables. The differences between the groups with high and low self-determination were further analyzed using the Chi-square test for categorical variables and the Mann-Whitney U test for quantitative variables. Exploration of the dimensions of experienced self-determination was presented in percentages. Descriptive statistics regarding self-rated quality of life were presented with median and quartiles and analyzed using the Mann-Whitney U test. Differences in medians and effect size measures [39] were used to quantify the differences in quality of life between the groups with higher versus lower self-determination. Reference measures for effect size give 0.2 for a small effect, 0.5 for intermediate effect, and 0.8 for a large effect [39]. The SPSS 22.0 for windows (SPSS Inc., Chicago, IL) was used for all statistical analyses. A *p*-value of *<* 0.05 was regarded as statistically significant.

### Ethical consideration

The study (Dnr 2016/04-31Ö) was approved by the Regional Ethics Review Board in Umeå, Sweden. Participants received information about the study and were assured of confidentiality in line with the Declaration of Helsinki. Participation was voluntary and written informed consent was collected from all participants. Participants could withdraw from the study or end their participation at any time without giving any reason.

## Results

Reported background characteristics (Table 1) show that 63% of the older adults receiving support from HCS were female. Participants had a mean age of 83 years (range 65–100, SD 7.9); 69% had received help from HCS for 1–10 years; 59% had one or more visits each day; 81% lived in an apartment; 48% had completed elementary school; 86% were born in Sweden; 93% spoke Swedish as their mother tongue; finally 95% were dependent in I-ADL to any extent and 83% were dependent in P-ADL to any extent. Background characteristics were similar in the two groups with higher and lower self-determination, except for type of housing. More adults who lived in a house with a garden reported higher self-determination (*p* = .034) than those who lived in an apartment (Table 1).

Overall, older adult’s self-reported self-determination varied between the dimensions (Table 2). A large proportion (72%) of the participants experienced self-determination in relation to the dimension use of time, followed by the dimensions self-care, mobility, social relationships, possibility to live as one wants, and activities. Finally, few people (15%) experienced self-determination in relation to the dimension help and support of others.

**Table 2:** Percentage of older adults (*n* = 136) who fully experienced self-determination

Dimensions	Self-determination, %
In relation to:	
Use of time (1 item)	72
Self-care (5 items)	70
Mobility (4 items)	51
Social relationships (5 items)	34
I live as I want (1 item)	34
Activities in and around the house (4 items)	33
Providing help and support for others (1 item)	15

In each dimension, older adults were considered to experience self-determination when they scored totally agree in all items within each dimension

Results show significant differences in QoL between the two groups of higher and lower experienced self-determination (Table 3). Older adults who reported a greater degree of self-determination reported a significantly higher experienced QoL on the EQ-5D-5 L total, the EQ-VAS and the NHP total score in comparison with those who reported a lower degree of self-determination. However, effect size measures show that the size of the differences can be considered low to intermediate. In sub-scales, older adults who reported a higher degree of self-determination also reported a higher degree on five out of six NHP sub-scales (*p* = 0.001–0.033) compared to adults who reported a lower degree of self-determination. In the sub-scale sleep, we could not find any differences (*p* = 0.395) between the groups.

**Table 3:** Differences between older adults who reported higher degree vs lower degree of self-determination on QoL

Health-related QoL	Higher self-determination Md (Q1; Q3)	Lower self-determination Md (Q1; Q3)	Differences in Median	Effect size	*p*
EQ-5D-5 L total^a^	0.8 (0.6; 0.9)	0.6 (0.3; 0.7)	0.2	0.39	< 0.001
EQ-VAS^a^	65 (50; 80)	50 (40; 65)	15	0.30	< 0.001
NHP total^b^	24 (8; 40)	40 (26; 53)	16	0.30	< 0.001

*n* difference between 117 and 128 depending on internal missing values

*p =* Differences between higher and lower self-determination analysed using Mann-Whitney U Test

EQ-5D-5 L total range between < 0 to 1. 1 = full health

EQ-VAS range 0–100. 100 = best imaginable health

NHP range from 0 to 100. 0 = best imaginable health

^a^ = Higher value = better QoL, ^b^ = Lower value = better QoL

## Discussion

This study explores the experienced self-determination among older adults and whether there are differences between groups with higher vs lower self-determination, and QoL. Our main finding shows that the majority of older adults with support from HCSs experienced self-determination in the dimensions use of time, and self-care. However, we found a large variation in self-reported self-determination between all dimensions. The results showed that the group with higher self-determination also reported a higher QoL as measured through the EQ-5D-5 L, EQ-VAS and NHP compared to those lower self-determination.

Findings where the majority of older adults experienced self-determination in the dimensions how to use their time as they wanted, and self-care, are comparable with another Swedish study which used the same questionnaire to measure self-determination [21]. Findings in the Swedish study found that over 90% of older adults experienced self-determination in the dimensions financial situation, use of time, and self-care. However, a larger proportion of their participants reported self-determination concerning all dimensions compared to our results. The same study also show that self-determination decreases when care needs increase. Their study included older adult with and without HCS and fewer people who were dependent in P-ADL and I-ADL [21], which might explain the differences between the two sets of results.

Our interpretation of many older adults reported self-determination in use of time and self-care is that those dimensions include things that are usually carried out within the home (deciding when to get washed, dressed, go to bed or the toilet etc.) where it might be easier to exercise control over them. In contrast, the findings where few people reported self-determination - mobility, social relationships, to live as I want, activities in and around the house, and having the possibility to help and support other people includes several domains where one is more dependent on other people and external factors. Dimensions that are carried out around the home and factors that must be planned for in collaborations with others might make older adults more dependent. Our interpretation is in line with [11, 40]. Their results show that perceived control over one’s life can be influenced by external factors (factors influenced by the outside world, others and systems).

The low frequency of older adults who experienced self-determination in the dimension social relationships is particularly worrying. One review [14] concludes that good relationships among the older person, staff, and healthcare providers are crucial for older adults to be in control and independent [14]. Further, social relationships are important for retaining mental and physical functions and QoL among older adults [20]. It has been found that professionals and older adults had different focuses at care planning meetings which might influence the social relationship between the older adult and healthcare providers [23]. Topics such as social situations and activities in daily life were mostly initiated by the professionals while questions about psychological and existential issues, such as death, meaningfulness and loneliness, were mostly initiated by the older adult or their family members.

Social relationships is closely related to relatedness which is one of three (autonomy, relatedness and competence) basic psychological needs highlighted in the self-determination theory [41]. Nursing homes accept that it is important to support these three basic psychological needs, in order to increase subjective wellbeing among frail residents. The three needs play a central role in nursing home interventions [42]. It is reasonable to interpret that the same applies to older adults receiving support from HCS.

### Methodological discussion and limitations

This cross-sectional study is a part of a larger project conducted in one municipality in northern Sweden, and contains the well-known limitations of cross-sectional studies regarding inability to detect important issues such as causality, trends and changes over time. However, in terms of generating hypotheses and detecting relationships for further study, cross-sectional studies can make a contribution [43], hopefully in this study as well. The local selection of data and the moderate response rate (50%) might increase the risk that the results are non-representative for others. As the survey included several questionnaires, older adults might have found it a burden to complete the survey which in turn might have influenced the response rate negatively. The IPA-O questionnaire included one question with low reliability “My chances to decide to go on the sort of trips and holidays I want to are good”, but was kept in the questionnaire because of the target group’s opinions in the face validity test and because the question is a part of the dimension mobility [9]. A majority of the participants in this study showed a dependency in I-ADL and with a relatively high number of missing data for the P-ADL. Considering these factors, it remains being difficult to draw much conclusions about associations between self-determination and dependency based on our data. It seems likely that these are complex associations that would be worthy of further evaluation in other contexts and samples.

## Conclusions

Results in the present study show a difference between the two groups of older adults; older adults who reported a higher degree of self-determination also reported significantly higher experienced QoL than the adults who reported lower degree of self-determination. Our results are not surprising as previous research in other areas has also found a positive relation between self-determination and QoL [2, 3]. However, as far as we know, this is the first study that focuses on this topic within HCS. Further studies into the relationship between self-determination, dependence and health-related QoL would be valuable, for example developing and testing models of care to strengthen the self-determination of older people in all aspects of HCS and to evaluate the impact of this on health-related QoL. Today, people are expected to live 15 to 20 years longer than previously in developed countries. Living longer might increase the length of time spent living with functional dependence, thus increasing the need for care models that also maintain self-determination and independence in respect to functional aspects are needed [44].

## Abbreviations

HCS: Home Care Service
IPA-O: Impact of participation and autonomy
NhP: Nottingham Health Profile
QoL: Quality of life
